# Case report: Dandy-Walker malformation with occipital encephalocele and superadded meningitis

**DOI:** 10.1016/j.heliyon.2025.e41883

**Published:** 2025-01-10

**Authors:** Saher Bano, Muhammad Aqib Faizan, Tooba Rehman, Jasleen Kaur, Jeevanjyot Singh

**Affiliations:** a Ayub Medical College, Khyber Medical University, Khyber Pakhtunkhwa, Pakistan; b Gomal Medical College, Khyber Medical University, Khyber Pakhtunkhwa, Pakistan; c Maharishi Markandeshwar Institute of Medical Sciences and Research, Ambala, India

## Abstract

Dandy-Walker malformation is a relatively uncommon abnormality known as cerebellar dysgenesis. This condition is characterized by cerebellar vermis hypoplasia, an upwardly rotated and malrotated vermis, an enlarged fourth ventricle, and an enlarged posterior fossa. This syndrome is thought to affect roughly one in every 35000 live births. Here we present a case of a preterm male of 33 + 4 weeks period of gestation who presented to us with a posterior midline cystic swelling at the occipital neck region. While there was no bruit in the transillumination of the cyst, there was a bruit in the transillumination of the skull.

Due to his grunting respiratory rate of 44 breaths per minute and heart rate of 140 breaths per minute at presentation, he was admitted to the newborn critical care unit. The occipital cyst was excised following neurosurgery on board after getting a CT scan brain done which showed dilated ventricles with normal pressure hydrocephalus, cystic cerebellar changes, communicating with 4th ventricle and occipital encephalocele. Brain parenchymal changes were also noted and a CSF R/E was sent which showed meningitis and the patient was put on IV antibiotics empirically along with fluid supplementation.

**Objective:**

This case report aims to highlight the clinical presentation, diagnostic approach, and management of Dandy-Walker Malformation (DWM) in a preterm neonate, emphasizing the importance of early recognition and intervention in similar cases.

**Rationale:**

This case underscores the need for careful evaluation of neonatal cystic lesions, particularly in preterm infants, where timely diagnosis and appropriate management can significantly affect outcomes. By documenting this case, we aim to contribute to the understanding of Dandy-Walker Malformation and improve clinical practices in neonatal care.

## 1 Introduction

**Fig. 1:**
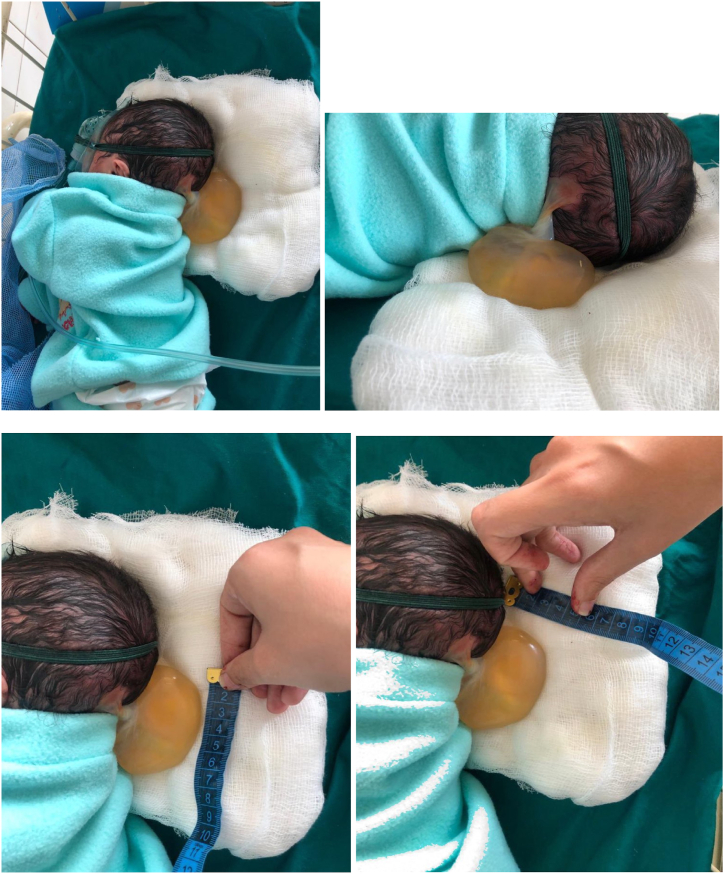
Multiple views of a large cystic mass in the occipital area that extends to the mid back, Lateral view, Posterior view, and Lateral view showing the approximate size of the cyst.

**Fig. 2:**
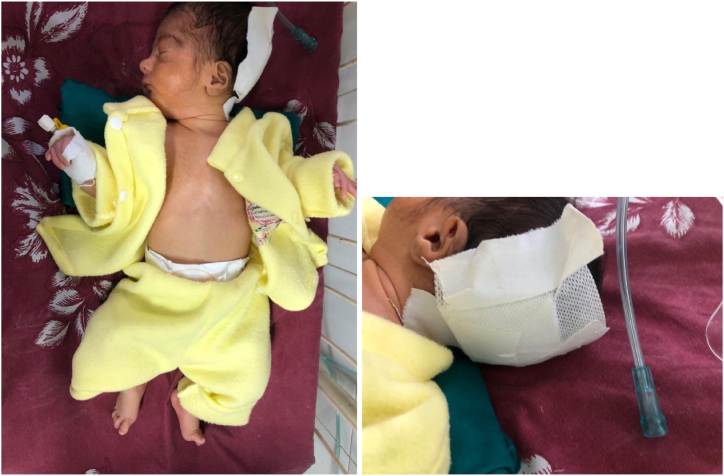
Lateral and posterior view of the patient showing surgical removal of the cyst.

**Fig. 3:**
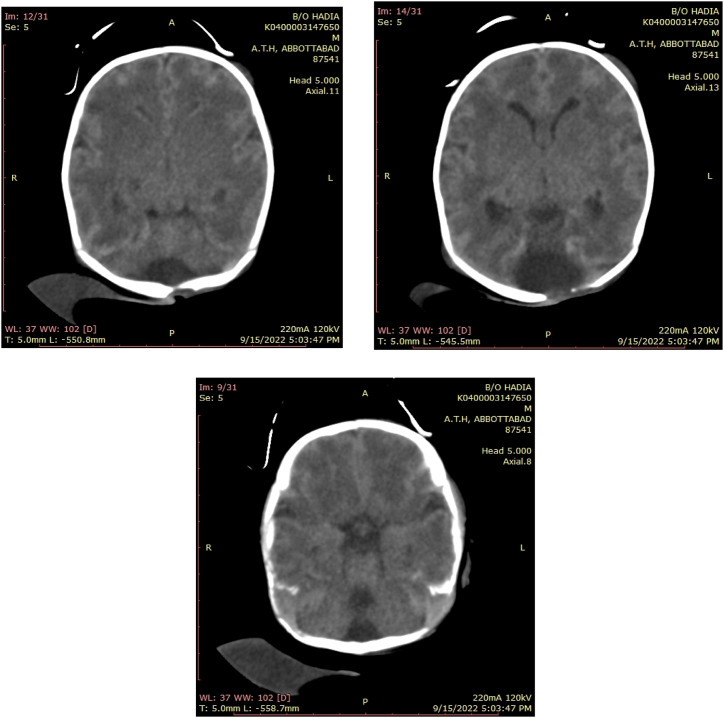
Post op CT scan.

One of the most common developmental abnormalities in the human brain, known as "Dandy-Walker Malformation," is characterized by cerebellar vermis hypoplasia, an upwardly rotated and malrotated vermis, an enlarged fourth ventricle, and an enlarged posterior fossa [1,2] (see Table 1, Table 2, Table 3, Table 4, Fig. 1, Fig. 2, Fig. 3).

**Table 1:** Clinical condition at presentation.

Vital Signs	Clinical Observations	Lab Tests
Weight:2kg	Not maintaining O2 Sats	Blood Screen
Temp: 98F	Normal Muscle Tone	FBC
R/R 44/min (Grunting)	Grunting ++	CT Brain Plain
HR 140/min	Poor Sucking reflex

**Table 2:** Clinical and diagnostic tests.

Clinical Tests	Hbs Ag -ve Anti-HCV -ve Anti-HIV -ve	Day 2 CBC WBC 11.2 Hb 15.2 HCT 44.4 PLT 215
Diagnostic Test	CT BRAIN PLAIN	Dilated lateral ventricles Dilated Cisterna Magna noted
Sampling Mechanisms	Blood tests via IV Line with 26G butterfly cannula	For the excision of the cyst, a neurosurgical opinion was taken and they took the pt. under their care for excision the pt. was handed over back to the Nursery after the excision

**Table 3:** Treatment Regular follow-ups with neonatologists, pediatricians, and neurosurgeons were recommended.

Drug	Route	Dose	Frequency	Opinion
Inf 10 % Dextrose	IV	30ml	QID	Neurosurgical opinion requested
Inj Ampicillin + Cloxacillin	IV	100mg	BID	
Inj Acyclovir	IV	40mg	TID

**Table 4:** Clinical condition of the patient post-treatment.

Vital signs	Clinical observations	Lab tests
Weight:1.75kg	Not maintaining O2 Sats	FBC
Temp: 98F	Normal Muscle Tone	CT Brain with contrast
R/R 40/min (Grunting)	Grunting (improved)	
HR 130/min	Poor Sucking reflex

Approximately one in every 35,000 live births is thought to have the Dandy-Walker Malformation and it is frequently linked to other congenital abnormalities, however, only about 34 cases of this syndrome have been reported to be connected with a massive occipital encephalocele [3]. To our knowledge which is based on a literature search on PubMed, Embase, and Cochrane, it is the first reported case of such anomalous association in Pakistan.

Another congenital neural tube defect is occipital encephalocele, which is marked by sac-like protrusions of the intracranial structures through the skull and Dural openings [4]. Based on the sac contents, it presents with different grades of neurologic impairments.

Prenatal ultrasound examination can diagnose both of these disorders while MRI is the preferred diagnostic test for post-natal diagnosis of occipital encephalocele [2,5].

Although the prognosis of this condition has improved with modern therapeutic approaches incorporating surgical procedures such as cranioplasty, ventriculoperitoneal shunts, and antibiotics, still the death rate remains high ranging from 26 to 50 % [[6], [7], [8]].

### 1.1 Case

A preterm male at 33 + 4 weeks of gestation, weighing 2 kg, was delivered via induced vaginal delivery (IOL) on September 14, 2022 at 7:00 p.m. in Abbottabad due to congenital malformations detected on prior sonography. The decision for induction was made due to suspicion of Dandy-Walker malformation. At birth, the neonate presented to the NICU with a cystic swelling protruding from the midline at the occipital and neck area.

APGAR scores were 10/10 at 0 minutes and 10/10 at 5 minutes. The infant cried immediately after birth, and resuscitation was not required.

At Presentation:

Maternal History: The mother had a history of Brucellosis and prior miscarriages:

• 1st Pregnancy: Miscarriage at 8 weeks EGA due to contracting Brucellosis.
• 2nd Pregnancy: Delivered at 34 weeks EGA. The baby had Truncus Arteriosus Type 1, a large VSD, and severe truncal regurgitation, presented with delayed cry and cyanosis at birth, and did not survive.
• 3rd Pregnancy: Miscarriage at 12 weeks EGA due to Brucellosis.
• 4th Pregnancy: Current pregnancy (G4P1) with induction at 33 + 4 weeks due to the suspicion of Dandy-Walker malformation.

After the third pregnancy, the mother completed treatment for Brucellosis with doxycycline and rifampin. Before conceiving again, her Brucella titers were checked and were found to be 1:80.

Prenatal Findings:

• Ultrasound detected oligohydramnios and cystic abnormalities in the area of the cisterna magna measuring 20mm × 17.5mm, communicating with the 4th ventricle.
• An extracranial cyst near the fetal neck, measuring 53mm × 68mm, was also noted.
• Ventricular dilatation and brain parenchymal thickness of 19 × 20.4mm were reported, suggesting macrocephaly.

Postnatal Presentation: The baby presented with an occipito-frontal circumference of 34cm and grunting at a respiratory rate of 44/min and a heart rate of 140/min. On examination at the NICU:

• The anterior fontanelle was wide open.
• A cystic swelling measuring approximately 7 × 4.5cm was noted at the occiput. The swelling did not change size with crying.
• Transillumination was positive in the cyst but negative in the skull.
A CT Brain showed dilated ventricles with normal pressure hydrocephalus, cystic cerebellar changes communicating with the 4th ventricle, and an occipital encephalocele [22].

The infant was diagnosed with Dandy-Walker malformation with occipital encephalocele. The occipital encephalocele was excised with the help of neurosurgery. The herniated gliosed non-viable portion of the brain was removed, and the Dural defect was repaired in a water-tight fashion. CSF analysis of the excised fluid showed evidence of meningitis.

Treatment: As the infant did not present with hydrocephalus, no shunting was performed. However, the baby was placed on IV Acyclovir (40mg TID), IV Ampicillin and Cloxacillin (100mg BD), and IV Cefotaxime (100mg BD) as per guidelines for meningitis. Supplemental oxygen inhalation was also administered for the grunting.

IV Line secured, NPO, Supplemental Oxygen started (SEPSIS PROTOCOL)

Grunting gradually improved by day 3 of admission. F/U CT head with contrast was ordered when the parents of the patient requested to shift the patient to a different hospital for follow-up and further management. The patient was moved to another hospital on supplemental oxygen support.

## 2 Discussion

The most prevalent congenital brain abnormality is known as Dandy-Walker Malformation which mainly affects the cerebellum as well as several of its other components such as the fourth ventricle, posterior fossa, and cerebellar vermis [9]. Numerous hypotheses have been put out to address the pathophysiology of this disease. The first theory was based on the hypothesis that the atresia of the Luschka and Magendie foramina happens in the eighth week of embryonic development [10].

This idea was later refuted because the cerebellar anlagen joined in the midline before the foramina became patent [11]. Benda [12]and others Hart et al. [13], and Golden et al. [14] believe this disease to be a complex developmental aberration, likely involving cerebellar cleaving and modifications to the fourth ventricle's membranes. At the same time, Brodal, and Hauglie-Hanssen [11], proposed it to be due to the primary anomaly is the early bulging of the rhomb encephalon's embryonic membranes before foramina form.

Although Dandy-Walker malformations are rare and can occur on their own, up to 90 % of cases are linked to other abnormalities including cardiac, gastrointestinal, orthopedic, genitourinary, and neurological conditions [15].50 % of patients have chromosomal abnormalities. Some may experience developmental problems [13,16]. Hart et al. [13] examined seven (25 %) of the cases with systemic defects, and Olson et al. [17]examined the seven reported cases with related heart malformations.

Although this syndrome is frequently linked to other CNS abnormalities, there aren't many documented cases of it being linked to occipital encephalocele. Of the 50 instances with Dandy-Walker Malformation that Bindal et al. [18] reported, eight had concomitant occipital meningocele. Although these authors could only locate 11 further cases in the global literature, they estimated that 16 % of DWM would have this connection [18]. Likewise, DWM is quite rare in large cephalocele series [[19], [20], [21]].In a set of 72 DWM, Mohanty et al. [22]did not find any OMC.

Only 34 examples of this syndrome linked to occipital encephalocele were found using the PubMed, Cochrane, and Medline databases. Early in the second trimester, ultrasonography or magnetic resonance imaging (MRI) can be used to identify DWM in a fetus [23].

Based on its clinical manifestation, which includes a tumor protruding through a bone defect in the skull, occipital encephalocele is diagnosed [5,24].

To stop more neurological deterioration, early treatment is essential [25]. To treat obstructive hydrocephalus, a surgical cystoloperitoneal (CP), ventriculoperitoneal (VP), or endoscopic third ventriculostomy (ETV) shunt must be installed [23].

The majority of patients have clinical signs of high intracranial pressure, typically brought on by a posterior fossa cyst or hydrocephalus. The most typical treatment for occipital encephalocele is surgical resection, as was done in our instance by neurosurgeons who removed it and sent a CSF R/E sample of the fluid, which revealed meningitis. The degree of the deformity and any related characteristics affect the patient's prognosis.

An excessive accumulation of fluid in and around the brain results from a blockage in the normal flow of cerebrospinal fluid; this increases intracranial pressure and head circumference and eventually causes neurological impairments. This explains the connection between Dandy-Walker deformity and hydrocephalus. Regrettably, since the infant in our instance did not exhibit hydrocephalus, shunting was not contemplated; however, as it might arise following surgical excision, routine monitoring was recommended. Almost all DWM and OCM patients noted shunting and hydrocephalus. For example, after OMC closure, numerous newborns acquired hydrocephalus rapidly even though it was not first apparent [18,26]. This illustrates the independent development of OMC and hydrocephalus.

## 3 Conclusion

The intricacy of treating such congenital abnormalities is highlighted by this case, which shows the uncommon co-occurrence of Dandy-Walker Malformation with occipital encephalocele and superadded meningitis. Improving outcomes for impacted patients requires early diagnosis and comprehensive therapy.

## Limitation

A key limitation in this case is that the patient's family chose to transfer the neonate to another facility of their own accord. As a result, we were unable to maintain contact and could not obtain follow-up information regarding the patient's subsequent care and outcomes.

## CRediT authorship contribution statement

**Saher Bano:** Writing – original draft, Project administration, Methodology, Investigation, Data curation. **Muhammad Aqib Faizan:** Writing – review & editing, Visualization, Validation, Supervision, Project administration, Conceptualization. **Tooba Rehman:** Writing – review & editing, Validation, Supervision, Project administration. **Jasleen Kaur:** Visualization, Validation. **Jeevanjyot Singh:** Visualization, Validation, Supervision.

## Ethics statement

Approval from the ethics committee was not required for this study. Consent for publication was directly obtained from the patient. The authors obtained permission from the hospital's director to utilize the patient's data and chest X-ray for research purposes.

## Declaration of competing interest

The authors declare that they have no known competing financial interests or personal relationships that could have appeared to influence the work reported in this paper.
